# Cross-Subject Seizure Detection in EEGs Using Deep Transfer Learning

**DOI:** 10.1155/2020/7902072

**Published:** 2020-05-08

**Authors:** Baocan Zhang, Wennan Wang, Yutian Xiao, Shixiao Xiao, Shuaichen Chen, Sirui Chen, Gaowei Xu, Wenliang Che

**Affiliations:** ^1^Chengyi University College, Jimei University, Xiamen 361021, China; ^2^Institute of Data Science, City University of Macau, Macau, China; ^3^School of Informatics, Xiamen University, Xiamen 361001, China; ^4^School of Electronics and Information Engineering, Tongji University, Shanghai 201804, China; ^5^Department of Cardiology, Shanghai Tenth People's Hospital, Tongji University School of Medicine, Shanghai 201804, China

## Abstract

Electroencephalography (EEG) plays an import role in monitoring the brain activities of patients with epilepsy and has been extensively used to diagnose epilepsy. Clinically reading tens or even hundreds of hours of EEG recordings is very time consuming. Therefore, automatic detection of seizure is of great importance. But the huge diversity of EEG signals belonging to different patients makes the task of seizure detection much challenging, for both human experts and automation methods. We propose three deep transfer convolutional neural networks (CNN) for automatic cross-subject seizure detection, based on VGG16, VGG19, and ResNet50, respectively. The original dataset is the CHB-MIT scalp EEG dataset. We use short time Fourier transform to generate time-frequency spectrum images as the input dataset, while positive samples are augmented due to the infrequent nature of seizure. The model parameters pretrained on ImageNet are transferred to our models. And the fine-tuned top layers, with an output layer of two neurons for binary classification (seizure or nonseizure), are trained from scratch. Then, the input dataset are randomly shuffled and divided into three partitions for training, validating, and testing the deep transfer CNNs, respectively. The average accuracies achieved by the deep transfer CNNs based on VGG16, VGG19, and ResNet50 are 97.75%, 98.26%, and 96.17% correspondingly. On those results of experiments, our method could prove to be an effective method for cross-subject seizure detection.

## 1. Introduction

Epilepsy, a disorder of normal brain function characterized by the existence of abnormal synchronous discharges in the cerebral cortex, impacts approximately 2% of the world's population and is likely to jeopardize their health and life. The epilepsy diagnose is always down by analyzing electroencephalogram (EEG), which includes scalp EEG and intracranial EEG. Scalp EEG signals have been wildly studied because they are relatively cheap and easy to gain. Commonly, 19 recording electrodes and a system reference are placed on the scalp area according to specifications by the International 10-20 system. For the seizure detection task, it is required to analyze the EEG signal thoroughly towards the decision of the existence of epileptic seizure or not, and this is an extensive clinical experience required and time-consuming job [1]. Due to the relatively infrequent nature of epileptic seizures, long-term EEG recordings are necessary, to make the situation of visually reading EEG signal by human experts worse. Therefore, computer-based technology for automatic seizure detection is urgently needed and is also a key method to save time and effort.

In previous studies, numerous detection algorithms have been proposed [2–4]. The features extracted from EEG signals come from time domain, frequency domain, time-frequency analysis [5, 6], wavelet analysis [7, 8], and so on. After the features are extracted, classifiers such as SVM and other machine learning algorithms are frequently used in the classification phase [9]. For example, Subasi et al. created a hybrid model to optimize the SVM parameters, showing that the proposed hybrid SVM model is a competent method to detect epileptic seizures using EEG [10].

In recent years, deep learning (DL) has been proven to be very successful in image classification, object detection, and segmenting, exhibiting near-human abilities to perform many tasks. DL extracts the global synchronization features automatically and does not need any a priori knowledge. Huang et al. proposed a coupled neural network for brain medical image [11, 12] and a deep residual segmentation network for analysis of IVOCT images [13]. Also, a number of recent studies demonstrated the efficacy of deep learning in the classification of EEG signals and seizure detection [14]. Convolutional neural network (CNN), as one of the most widely used deep learning models, is always used. For example, Wang et al. proposed a 14-layer CNN for multiple sclerosis identification [15]. For the seizure detection task, there are two ways of using the original EEG signals as the input image of CNN. On one hand, segments of raw EEG signals with different lengths serve as input directly. Emami et al. divided EEG signals into short segments on a given time window and converted them into plot images; each of which was classified by VGG16 as “seizure” or “nonseizure” [16]. Their experiments resulted in that the median true positive rate of CNN labeling was 74%. On the other hand, time and frequency domain signals extracted from raw EEG signals serve as input image of CNN. Zhou et al. designed a CNN with no more than three layers to detect seizure, with time-frequency spectrum image as input and achieved average accuracy 93% [17]. They also compared the performance of time domain with that of time-frequency domain, which resulted that frequency domain signals have greater potential than time domain signals for CNN applications. Although, deep learning especially CNN has made remarkable progress in the field of EEG classification and seizure detection, the performance of seizure detection still requires improvement. The challenge comes from two aspects. Firstly, training a deep learning model such as VGG16 needs a large amount of labeled data. However, most of the EEG signals data are unlabeled. In this study, the public-labeled scalp EEG dataset from the Children's Hospital Boston-Massachusetts Institute of Technology (CHB-MIT, see http://physionet.org/physiobank/dataset/chbmit [18]) will be used as the raw signals. Due to the relatively infrequent nature of epileptic seizures, the raw signals will be augmented to avoid extremely unbalanced dataset for training. Secondly, EEG signals are person-specific. On the other hand, Orosco proposed a patient nonspecific strategy for seizure detection based on stationary wavelet transform of EEG signals and reported the mean sensitivity of 87.5% and specificity of 99.9% [19]. Hang et al. proposed a novel deep domain adaption network for cross-subject EEG signal recognition based on CNN and used the maximum mean discrepancy to minimize the distribution discrepancy between source and target subjects [20]. Akyol presented a stacking ensemble based deep neural network model for seizure detection. Experiments were carried out on the EEG dataset from Bonn University and came to the result that the average accuracy is 97.17% along with average sensitivity of 93.11% [21]. Zhang et al. proposed an explainable epileptic seizure detection model to the pure seizure-specific representation for EEG signal through adversarial training, in order to overcome the discrepancy of different subjects [22].

CNN models like VGG16 have millions of parameters to be trained, not to mention deeper network like googLeNet. Transfer learning has emerged to tackle this problem, especially in real-world applications. Transfer learning is always down by a pretrained model, which is trained on the benchmark dataset like ImageNet. The pretrained model can extract universal low-level features of images and can tremendously improve the efficiency of using CNN. However, the pretrained model should be fine-tuned in order to match the target dataset and its goal. For example, Shao et al. created a deep transfer CNN for fault diagnosis, in which a pretrained CNN model is used to accelerate the training process [23].

**Figure 1:**
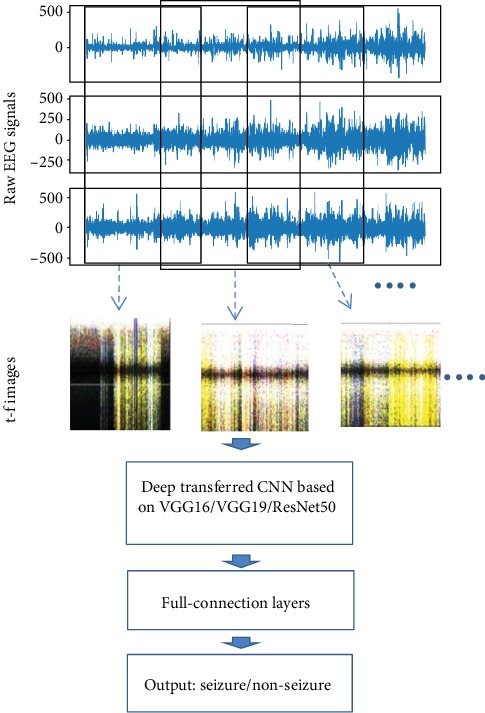
The flow of seizure detection.

In this paper, three transfer CNN models, based on VGG16, VGG19, and ResNet50, respectively, are proposed for seizure detection. The flow of seizure detection is shown in Figure 1. Let us take VGG16 as an example. The target CNN network consists of a pretrained VGG16 model with nontop layers frozen and fine-tuned top layers. The pretrained model uses parameters based on ImageNet. The fine-tuned top layers include an output layer with two categories: seizure and nonseizure.

For experiments, the public CHB-MIT EEG dataset is used. Raw EEG signals from FP2-F8, F8-T8, and T8-P8 electrodes are converted to time-frequency spectrum image using short-time Fourier transform (STFT) [24] and then fused as one image, inspired by [25]. The fused images from different persons all putted together are the target dataset. Then, the target dataset serves as the input of the deep transfer models, to perform cross-subject seizure detection.

The remainder of this paper is organized as follows.  Section 2 introduces the used dataset from CHB-MIT.  Section 3 describes in detail the conversion process of EEG signal to time-frequency spectrum image and introduces all three deep transfer models.  Section 4 conducts experiments and gives a comparison of three models.  Section 5 discusses the results.  Section 6 concludes the paper with a summary.

## 2. Dataset Description

The dataset used in this paper is an open-source EEG database from the MIT PhysioNet, collected at the Children's Hospital Boston (CHB-MIT). The dataset consists of recordings from pediatric subjects with intractable seizures using scalp electrodes. Recordings are grouped into 23 cases. Each case contains 9 to 42 hours' continuous recordings from a single subject. All subjects were asked to stop related medical treatments one week before data collection. The sampling frequency for all subjects was 256 Hz. The start time and end time of epileptic seizure were labeled explicitly based on expert judgments. Most recordings contain 23 EEG channels and multiple seizure occurrences.

The duration of seizure varies for each subject very much. Thus, some of the recordings with relatively long seizure durations are used only. The reason for not including all the recordings is that recordings with a low proportion of seizure duration lead to an unbalanced dataset and would cause over-fitting of the CNN model.

## 3. Methods

**Figure 2:**
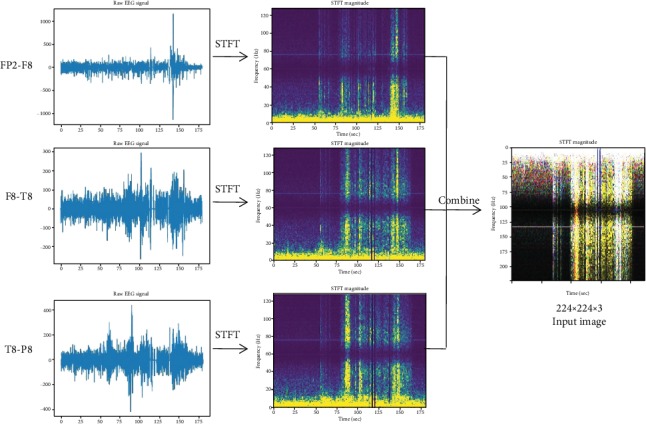
The conversion process from raw EEG signals to time-frequency image. In CHB-MIT EEG database, segments of raw signal from subject No. 13 are converted to t-f images by using STFT. Then, three t-f images are treated as three channels to form an input image.

In this section, we convert the raw EEG signals to time-frequency (t-f) spectrum images by using STFT and combine three t-f images from different channels as one input image, as shown in Figure 2. Then, deep transfer CNNs are proposed for seizure detection.

### 3.1. Data Preparation Based on STFT

The short-time Fourier transform (STFT) is used to analyze how the frequency content of a nonstationary signal changes over time. The STFT of a signal is calculated by sliding a window over the signal and calculating the discrete Fourier transform of the windowed signal. The window moves along the time axis at a given interval, with overlap or not. Commonly, overlap is used in order to compensate for the signal attenuation at the window edges.

Formally, STFT is defined as follows:

(1) $$\begin{array}{c} \text{STFT}\left(\tau ,\omega \right)=\int_{-\infty }^{+\infty }x\left(t\right)h\left(t-\tau \right)e^{-j\omega \;t}d\;t, \end{array}$$

where *x*(*t*) is the original EEG signal, *h*(*t*) is a window function, and *τ* is the window position on time axis.

Although, epileptic seizure is person-specific, they could have something in common. According to [24], signals from FP2-F8, F8-T8, and T8-P8 electrodes are relatively prominent. In this paper, we use signals from those three electrodes. And the t-f images from FP2-F8, F8-T8, and T8-P8 are treated as red, green, and blue channels, respectively, while combining them as one input image.

**Figure 3:**
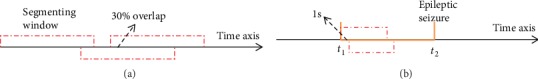
(a) Segmenting the signal of EEG along the time axis with 30% overlap and (b) segmenting the signal with one second per step in time interval [*t*_1_, *t*_2_], while epileptic seizure happening.

Due to the infrequent nature of seizures, the number of positive samples should be increased in order to avoid unbalanced dataset by augmenting. In detail, we prepare the dataset by two steps. Step one, as shown in Figure 3(a), for each signal of EEG, we move a window of length 180 seconds along the time axis with 30% overlap. Then for each windowed segment, we use STFT to calculate complex amplitude versus time and frequency, where window used by STFT is 413 signal points long and is moved along the time axis with 50% overlap. This results in a spectrum image whose size is 207 × 224, where 207 is the number of sample frequency and 224 the number of segment times. At last, the spectrum image is resized to 224 × 224. When epileptic seizure happens in the windowed segment, the resized spectrum image is labeled positive, otherwise negative. Step two, as shown in Figure 3(b), for each signal of EEG, let us assume that epileptic seizure happens at time interval [*t*_1_, *t*_2_]. Let the segmenting window start at *t*_1_ and move along the time axis one second per step, and furthermore overlap with [*t*_1_, *t*_2_] no less than 3 seconds. Then, each segmented window is converted to a spectrum image of size 224 × 224 as in step one. All spectrum images in this step are labeled positive.

In order to avoid an unbalanced dataset, only the EEG signals of subjects No. 05, 08, 11, 12, 13, 14, 15, 23, and 24 with relative long duration of epileptic seizure are used. This results in the target dataset of 8474 images of size 224 × 224 × 3, of which about 49.5% are labeled positive.

### 3.2. Deep Transfer Model

A pretrained model is a saved network that was previously trained on a huge dataset, such as ImageNet. Then, we can use transfer learning to customize this model to a given task. Intuitively, if a model is trained on a large and general dataset, this model could extract low-level features and serve as a generic model of the visual world. Then, we could use this model to extract meaningful features from new samples and add a new classifier on top of it to do specific classification, where only the added layers should be trained from scratch on our dataset. Transfer learning has also been proved effective in applications [26, 27].

**Figure 4:**
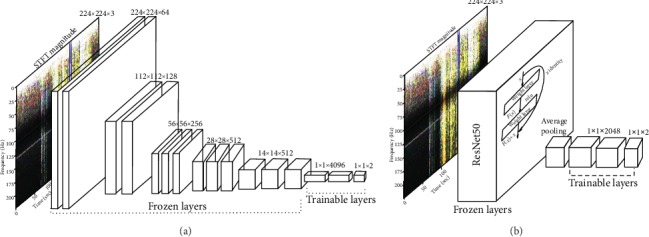
(a) The deep transfer model from VGG16, where the output layer is replaced by a new softmax output layer with two neurons, corresponding to seizure or nonseizure. (b) The deep transfer model from ResNet50, where the output layer is replaced by a new softmax output layer with two neurons, corresponding to seizure or nonseizure, and the size of full connection layers are also altered.

In this paper, three deep transfer models are proposed for comparison:

1. The deep transfer model based on VGG16 (referred as Model-1): deep neural network VGG16 is a well-known CNN model with 16 layers introduced in 2014 and has achieved amazing performance in various image tasks. VGG16 is characterized by small-sized filters, which is very suitable for our purpose of detecting the difference in frequency between seizure and nonseizure. As can be seen in Figure 4(a), Model-1 consists of a transferred VGG16 and top trainable layers. The transferred VGG16, with the output layer removed from the original model, is pretrained using the ImageNet database. The added top trainable layers consist of two trainable full connection layers and a softmax output layer with two neurons corresponding to seizure or nonseizure.
2. The deep transfer model based on VGG19 (referred as Model-2): Model-2 has almost the same structure as Model-1. The only difference between those two transfer models is that one use pretrained VGG16 and the other use pretrained VGG19.
3. The deep transfer model based on ResNet50 (referred as Model-3): ResNet50 is one of the famous residual neural networks, which are characterized by utilizing shortcuts to jump over some layers. As shown in Figure 4(b), the first part of Model-3 is a pretrained ResNet50 without top layers. Then after global average pooling, two full connection layers of 2048 neurons are added. The output is a softmax output layer with two neurons.

The loss function used in three models is softmax cross entropy, which is defined as

(2) $$\begin{array}{c} H\left(r,p\right)=-\underset{i}{\sum }r_{i}\cdot \log\left(p_{i}\right) \end{array}$$

where *r* and *p* are the labeled and predicted probabilities, respectively.

## 4. Experiments and Results

Experiments have been carried out to evaluate the performance of the proposed deep transfer models. The test platform is a desktop system with Nvidia RTX 2080Ti and 64GB memory running Ubuntu.

### 4.1. Training the Deep Transfer Network

After being shuffled, the target datasets are divided into training set, validation set, and test set, which occupy 60%, 20%, and 20%, respectively.

Model-1 and Model-2 almost have the same structure, so their training methods are of the same. Let us take Model-1 for example. The parameters of VGG16 pretrained on ImageNet are transferred to the network and would be frozen while training. Other trainable parameters are initialized randomly. The optimizer is SGD with learning rate of 0.001 and a small decay of 1*e*-5. Then, the network is trained and validated on the training set and validation set, respectively, with batch size of 64. As for Model-3, we transfer the parameters of ResNet50 pretrained also on ImageNet and froze them through training. Other trainable parameters are also initialized randomly. For the training optimizer, the Adam algorithm is used, with a starting learning rate of 0.001, beta_1 of 0.9, and beta_2 of 0.999. The learning rate would be reduced by a factor 0.8 when validation loss has stopped descending for 5 epochs. Then, the network is trained and validated on the training set and validation set, respectively, with a batch size of 16. For all three models, the epoch is set to be 500, but the training would be stopped while validation loss is not descending for 20 epochs.

### 4.2. Results and Analysis

The statistical measures for evaluating classification performance contain accuracy (acc), sensitivity (recall), precision, and the Matthews correlation coefficient (mcor). The measure mcor takes into account true and false positives and negatives and is regarded as a balanced measure. The more mcor approaches 1, the better the prediction is. Formally, their definitions are as follows:

(3) $$\begin{array}{c} \text{accuracy}=\frac{TP+TN}{N},\\ \text{precision}=\frac{TP}{TP+FP},\\ \text{recall}=\frac{TP}{TP+FN},\\ \text{mcor}=\frac{TP\times TN-FP\times FN}{\sqrt{\left(TP+FP\right)\left(TP+FN\right)\left(TN+FP\right)\left(TN+FN\right)}}, \end{array}$$

where TP means the true positive, TN the true negative, FP the false positive, FN the false negative, and *N* the total.

**Figure 5:**
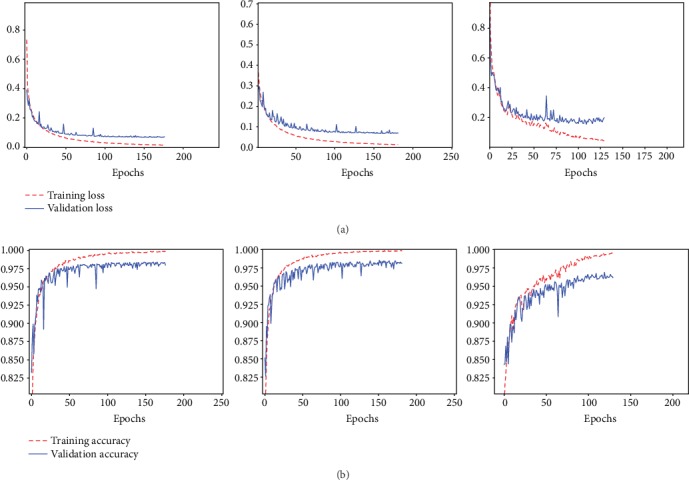
From left to right (with full connection size of 4096 × 4096, 4096 × 4096, and 2048 × 2048, respectively). (a) The loss of transferred mode based on VGG16, VGG19, and ResNet50. (b) The accuracy of transferred mode based on VGG16, VGG19, and ResNet50.

As shown in Figure 5, both the loss and accuracy of Model-1 and Model-2 converge after about 170 epochs. The loss and accuracy of Model-3 converge after about 100 epochs. But the metrics, including loss and accuracy, of deep transfer networks based on VGG16 and VGG19 are better than those of the deep transfer network based on ResNet50. Because the test dataset is randomly selected from the target dataset, the training and testing processes are carried out 10 times, and then, the average of all metrics is used. As shown in Table 1, the performance of Model-1 is almost the same as that of Model-2. But both models based on VGG outperform the model based on ResNet50 (Model-3).

For comparison, different sizes of full connection layers of each model are used for seizure detection. All experiments are carried out with the same training, validating and testing datasets. The results are shown in Table 2.

## 5. Discussion

For seizure detection, the methods frequently used include classical ones like SVM and modern ones like convolutional neural networks. Conventional approaches rely on time and frequency, where methods based on frequency are more capable and efficient than on time. But the frequency bands should be customized for a particular patient, which makes it very difficult to generalize this method to different patients. The latest CNNs used in this study are highly suited for EEG classification because they can select features adaptively and automatically. In order to take the advantage of frequency, we use the time-frequency images as an input dataset of the CNNs.

The prominent CNN like VGG16 has tens of millions of parameters. If all those parameters are trained from scratch, millions of images would be needed to ensure that the network could select features properly. The demand of so many images could be almost impossible to meet due to the infrequent nature of epileptic seizure. On the other hand, the images from ImageNet and our time-frequency images would have low-level universal features in common. So, we transfer the parameters pretrained on ImageNet to our models, to extract universal features, and train from scratch the parameters of the full connection layers.

Most existing literatures on seizure detection are patient-specific, which require a priori knowledge of the patient. In this study, the deep transfer models, by using the CHB-MIT EEG dataset as the original signals, are patient-independent. The t-f images from different objects are put together and shuffled, from which the testing dataset is selected randomly. For comparison, three transfer models based on VGG16, VGG19, and ResNet50, respectively, are proposed. Experiments are carried out to evaluate their performance of cross-subject seizure detecting. In detail, the input dataset, generated from the original signals, consists of 8474 t-f images. The ratio of positive to negative samples is almost one to one after augmentation. Then, all three transfer models are trained, validated, and tested on our augmented dataset. The average accuracies are 97.95%, 98.26, and 96.17%, respectively.

However, in the original EEG dataset, not all signals are used, due to the short durations of epileptic seizure. Those cases are not rare. So, a method for seizure detection including those objects will be addressed in the future. GAN might be a choice to tackle the problem, with its utilization of generative model.

## 6. Conclusions

This study gives a method to detect seizure in EEGs for cross-subjects, by using deep transfer learning. Three deep transfer models are proposed based on VGG16, VGG19, and ResNet50, respectively. Experiments are performed to evaluate the models over the CHB-MIT EEG dataset, without the need for denoising the EEG signals. Also, on the same dataset, experiments of the three models with full connection layers of different sizes are carried out for comparison. In the future, we plan to extend this method to EEG signals with a relatively short duration of epileptic seizure.

## Figures and Tables

**Table 1:** The test loss, accuracy, and metrics of recall, precision, and mcor (average).

Deep transfer network	Loss	Accuracy	Recall	Precision	Mcor
Model-1 (on VGG16)	0.0659	0.9795	0.9844	0.9747	0.9589
Model-2 (on VGG19)	0.0699	0.9826	0.9801	0.9851	0.9653
Model-3 (on ResNet50)	0.2249	0.9617	0.9865	0.9400	0.9246

**Table 2:** The metrics of each model with different full connection size.

Size of FC	Base model	Loss	Accuracy	Recall	Precision	Mcor
2048 × 2048	Model-1	0.0631	0.9837	0.9837	0.9839	0.9674
	Model-2	0.0698	0.9816	0.9844	0.9789	0.9632
	Model-3	0.1790	0.9578	0.9596	0.9563	0.9157
1024 × 1024	Model-1	0.0638	0.9826	0.9844	0.9810	0.9653
	Model-2	0.0731	0.9805	0.9823	0.9788	0.9611
	Model-3	0.1739	0.9663	0.9806	0.9526	0.9331
4096 × 2048	Model-1	0.0596	0.9833	0.9823	0.9844	0.9667
	Model-2	0.0712	0.9791	0.9851	0.9734	0.9583
	Model-3	0.1820	0.9568	0.9724	0.9430	0.9140
2048 × 1024	Model-1	0.0618	0.9830	0.9851	0.9810	0.9660
	Model-2	0.0690	0.9815	0.9844	0.9789	0.9631
	Model-3	0.2020	0.9567	0.9752	0.9406	0.9142
4096 × 1024	Model-1	0.0637	0.9816	0.9851	0.9782	0.9631
	Model-2	0.0672	0.9808	0.9851	0.9768	0.9617
	Model-3	0.2091	0.9564	0.9752	0.9400	0.9135
1024 × 512	Model-1	0.0673	0.9809	0.9837	0.9782	0.9618
	Model-2	0.0758	0.9777	0.9872	0.9687	0.9555
	Model-3	0.1649	0.9642	0.9851	0.9456	0.9292
1024 × 256	Model-1	0.0624	0.9844	0.9823	0.9865	0.9688
	Model-2	0.0664	0.9808	0.9872	0.9748	0.9618
	Model-3	0.1452	0.9610	0.9653	0.9572	0.9221
512 × 512	Model-1	0.0678	0.9769	0.9851	0.9693	0.9541
	Model-2	0.0669	0.9815	0.9851	0.9782	0.9632
	Model-3	0.1201	0.9773	0.9879	0.9674	0.9548
512 × 256	Model-1	0.0653	0.9794	0.9844	0.9747	0.9589
	Model-2	0.0841	0.9720	0.9880	0.9575	0.9445
	Model-3	0.1198	0.9745	0.9844	0.9653	0.9491

## Data Availability

The data used to support the findings of this study are available from the corresponding author upon request.
